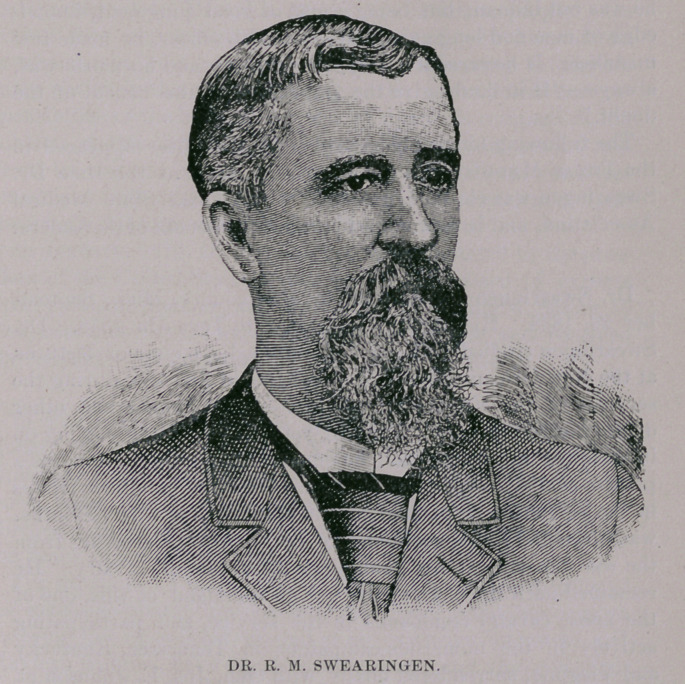# Death of Dr. Swearingen

**Published:** 1898-09

**Authors:** 


### Death of Dr. Swearingen.

DR. RICHARD MONTGOMERY SWEARINGEN, State Health Officer and ex-Officio Surgeon-General of Texas, died of Bright's disease at his home in Austin, Texas, Sunday, August 7 (ult.), at 4 p. m., aged 59 years, 10 months and 12 days.

Dr. Swearingen was an A. M. of Centenary College, Mississippi (1859), a graduate of medicine of the New Orleans School of

Medicine of the Class of 1867. In 1886 he received the honorary degree M. D., conferred on him by the Louisville Medical College.

He had a national reputation as a sanitarian, having held the office of State Health Officer from 1881 continuously, with the exception of four years under Governor Ross, to the day of his death—a service of fifteen years and six months. His long

DR. R. M. SWEARINGEN.

training in the office made him an ideal health officer, and his loss to Texas and to the South is felt by all classes.

Dr. Swearingen was a man of exceptionally fine character and attainments—both literary and professional. His personal popularity was something remarkable. He had a host of warmly attached friends, and was greatly beloved for his worth. As a physician he was eminent and successful; as an orator he was eloquent and gifted. He wrote little for the press, but what he wrote was always forceful, clear and to the point. His record as a Confederate soldier is matter of history. He had great courage,—morally and physically,—and he did his duty as he conceived it, without regard to the consequences to anybody or anything other than—and always with an eye single to, the public health. No amount of pressure—either political or commercial—could move him to depart one hair's breath from the line of duty as he saw it. To say that he was not infallible it to say he was but human; but being a man of good judgment, knowledge of men and long experience as health officer, he made few mistakes. If he erred it was on the safe side, and his motto was, always—"If in doubt give the public health the benefit of the doubt."

The following biographical sketch by the editor appeared in the TEXAS MEDICAL JOURNAL in May, 1893, at the time Dr. Swearingen was elected President of the Texas State Medical Association, and is reproduced here for the benefit of its readers:

Dr. Swearingen was born in Noxubee county, Miss., September 26, 1838. His parents emigrated to Texas in 1848. Dr. Swearingen studied medicine and attended a course of lectures at the New Orleans School of Medicine in 1859-60, during the services of the Flints—Sr. and Jr.—E. D. Fenner and other distinguished teachers. The war between the states coming on interrupted his studies and he entered the Southern army as a private soldier, responding to the first call for volunteer troops in Texas, February 28, 1861, and in the course of a few months, was promoted to the command of his company, receiving from the war department his commission as captain of cavalry. He remained until the close of the war in command of this, one of the finest cavalry commands in the service, and participating actively in the numerous campaigns in Tennessee, Kentucky and Virginia; surrendered finally with Gen. Jos. E. Johnson at Charlotte, N. C., when resistance was no longer possible.

During the war he married Miss Jennie Jessee, the daughter of a Tennessee gentlemen, at whose house the doctor was left sick on one occasion.

Upon the cessation of hostilities he returned to Texas, and locating in Washington county, in the town of Chapel Hill, he resumed the study of medicine with Dr. Rogers, and, attending a second course of lectures in 1867 at the New Orleans School of Medicine, was graduated M. D., with first honors, delivering the valedictory of his class.

Engaging immediately in the practice of his profession at Chapel Hill, Dr. Swearingen commanded at once a large practice. In the spring of 1875 he removed to Austin, where he made his residence up to the time of his death.

In the yellow fever epidemic of 1878, Dr. Swearingen and Dr. T. D. Manning volunteered their services to the Howards and proceeded to Memphis to aid the stricken people. On arrival they were assigned to Holly Springs, with instructions to take charge and establish a hospital. Manning falling an early victim in the unequal struggle, the entire labor and responsibility of the hospital service fell upon his companion, the subject of this sketch. The history of those sad scenes will never be written, but the services of these two Texas volunteer physicians, both of whom are dead, are indellibly engraved upon the hearts and memories of a grateful community.

In January, 1879, Dr. Swearingen was appointed by the president of the United States a member of the board of experts upon epidemic diseases, and assisted in preparing a report to Congress that met the expectations of the people and received the unqualified endorsement of the medical profession.

In February, 1881, he was appointed by Governor O. M. Roberts State Health Officer of Texas. He was reappointed by Governor Ireland in 1884 and served four years. He did not serve under the Ross administration, but was appointed by Governor Hogg in 1892, serving four years. Governor Culberson reappointed him in 1895.

For fifteen and a half years the control and administration of the extensive quarantine system oi the State, extending over an immense line of seacoast, as well as an extensive interstate and Mexican frontier, have devolved upon him, and the conspicuous exemption of the Texas people from epidemic disease during that time, as well as his reappointments testify to the distinguished ability with which his trusts have been discharged. In addition to his official duties as stated, Dr. Swearingen was constantly engaged in a general practice, yet he found time for social enjoyment, into which he entered with all the zest of an intense Southern nature, and also to contribute to the literature of his profession, he being an active member of the County,

State and National Medical Associations, and was appointed several years ago by the executive committee of the International Medical Congress to an important position on the Section of State Medicine and Public Hygiene, a subject to which he had given much attention. He was regarded as one of the foremost and most enlightened sanitarians of this progressive age.

Among his literary productions, his eulogy on the life and services of Dr. T. D. Manning, delivered as essayist for the occasion, before the Texas State Medical Association at Belton in 1884, stands conspicuous as a model of eloquence and pathos. It will go on record as one of the most chaste and merited tributes ever paid to departed worth.

At the meeting of the Texas State Medical Association, held in San Antonio in 1889, he was unanimously elected president.

## Figures and Tables

**Figure f1:**